# A Reinforcing Circuit Action of Extrasynaptic GABA_A_ Receptor Modulators on Cerebellar Granule Cell Inhibition

**DOI:** 10.1371/journal.pone.0072976

**Published:** 2013-08-19

**Authors:** Vijayalakshmi Santhakumar, Pratap Meera, Movses H. Karakossian, Thomas S. Otis

**Affiliations:** 1 Department of Neurobiology, David Geffen School of Medicine, University of California Los Angeles, Los Angeles, California, United States of America; 2 Department of Neurology and Neurosciences, Rutgers New Jersey Medical School, Newark, New Jersey, United States of America; McLean Hospital/ Harvard Medical School, United States of America

## Abstract

GABA_A_ receptors (GABARs) are the targets of a wide variety of modulatory drugs which enhance chloride flux through GABAR ion channels. Certain GABAR modulators appear to acutely enhance the function of δ subunit-containing GABAR subtypes responsible for tonic forms of inhibition. Here we identify a reinforcing circuit mechanism by which these drugs, in addition to directly enhancing GABAR function, also increase GABA release. Electrophysiological recordings in cerebellar slices from rats homozygous for the ethanol-hypersensitive (α6100Q) allele show that modulators and agonists selective for δ-containing GABARs such as THDOC, ethanol and THIP (gaboxadol) increased the frequency of spontaneous inhibitory postsynaptic currents (sIPSCs) in granule cells. Ethanol fails to augment granule cell sIPSC frequency in the presence of glutamate receptor antagonists, indicating that circuit mechanisms involving granule cell output contribute to ethanol-enhancement of synaptic inhibition. Additionally, GABAR antagonists decrease ethanol-induced enhancement of Golgi cell firing. Consistent with a role for glutamatergic inputs, THIP-induced increases in Golgi cell firing are abolished by glutamate receptor antagonists. Moreover, THIP enhances the frequency of spontaneous excitatory postsynaptic currents in Golgi cells. Analyses of knockout mice indicate that δ subunit-containing GABARs are required for enhancing GABA release in the presence of ethanol and THIP. The limited expression of the GABAR δ subunit protein within the cerebellar cortex suggests that an indirect, circuit mechanism is responsible for stimulating Golgi cell GABA release by drugs selective for extrasynaptic isoforms of GABARs. Such circuit effects reinforce direct actions of these positive modulators on tonic GABAergic inhibition and are likely to contribute to the potent effect of these compounds as nervous system depressants.

## Introduction

GABA_A_ receptors (GABARs), the main class of inhibitory neurotransmitter receptors, are pentameric ion channels composed from combinations of 19 subunits. Two broad categories of GABARs, synaptic and extrasynaptic, can be distinguished on the basis of molecular makeup, localization relative to synapses, and functional properties. Synaptic GABARs mediate fast phasic signaling and are made up of 2α, 2β and a γ subunit, a subunit composition associated with low GABA affinity and high efficacy. Extrasynaptic GABARs are formed by α4 or α6 and in some cases α1 subunits partnering with δ rather than γ subunits or GABARs with α5 subunits [1]. Extrasynaptic GABARs are excluded from postsynaptic densities and exhibit high GABA affinity and low desensitization, allowing them to generate tonic inhibition, which exerts a powerful influence on the excitability of certain classes of neurons [2], [3].

Extrasynaptic GABAR isoforms are modulated by a diverse set of sedative and anesthetic compounds [1], [4]. Low nanomolar concentrations of endogenous neurosteroids such as THDOC, act on GABARs containing δ subunits and have anesthetic actions [5]–[7]. Several general anesthetics like propofol and isoflurane are known to enhance tonic GABA currents [8], [9]. THIP (gaboxodol), a specific agonist at δ-containing GABARs at low concentrations [10]–[12] has been under clinical trial for insomnia [13]. Ethanol, the most common recreational neurodepressive drug causes robust, dose dependent increase in tonic GABA currents [14]–[18].

Modulators at extrasynaptic GABARs have also been shown to enhance synaptic GABA release. Recent studies have demonstrated that the frequency of GABAergic inputs to dopaminergic neurons in the ventral tegmental area is enhanced by THIP and that this enhancement is blocked by furosemide, an antagonist of extrasynaptic GABARs with α6 subunits [19]. In the cerebellum, the presynaptic effect of GABA modulators has been most clearly demonstrated with ethanol. Robust ethanol-induced increases in GABA release are observed at Golgi cell to granule cell synapses [15], [18]. Recent studies have identified that ethanol can increase Golgi cell firing in the presence of synaptic blockers indicating a direct effect [20], [21]. However, studies on rat lines homozygous for either the normal (α6100R) or the ethanol-hypersensitive (α6100Q) allele of the extrasynaptic α6 subunit gene have shown genotype-specific increases in ethanol-potentiation of granule cell sIPSC frequency in α6^100Q/100Q^ rats [18]. Since GABARs with α6 and δ subunits uniquely underlie tonic GABA currents in cerebellar granule cells [5], [22]–[24], α6-dependent effects imply that ethanol, and potentially other modulators of tonic GABA currents, indirectly modify GABA release onto granule cells by changing activity in the circuit. Here we used ethanol-hypersensitive (α6100Q/Q) rats, and wild-type and GABAR δ subunit knockout (*Gabrd-/-*) mice to test the hypothesis that circuit mechanisms play a fundamental role in this enhancement of granule cell synaptic inhibition. The findings suggest common modes of pre- and postsynaptic action for neurodepressants that enhance tonic GABA currents.

## Materials and Methods

### Electrophysiology

Parasagittal slices of the cerebellum (300 µm) from 21- to 40 day old Sprague Dawley rats homozygous for α6^100Q^ subunits, adult C57BL/6 (Jackson Laboratories, Bar Harbor, ME, USA) and *Gabrd*–/– mice lacking the GABAR δ-subunit on C57BL/6 background (Jackson Laboratories, Bar Harbor, ME, USA; generously provided by Dr. Istvan Mody at the University of California, Los Angeles) were obtained from animals sacrificed under deep halothane anesthesia [18], [25]. A group of C57BL/6 mice were genotyped using real time PCR with specific probes designed for each allele (Transnetyx, Cordova, TN). All mice tested (n = 10 mice) were found to be homozygous for the α6^100R^ allele. Surgical anesthesia was verified by the absence of response to hind limb pinch. All animal procedures were in accordance with protocol No.: 1998-139-42, approved by the University of California at Los Angeles Chancellor’s Animal Research Committee (IACUC No.: A-3196-01) and protocol No.: 12094 approved by the University of Medicine and Dentistry of New Jersey, Newark, NJ, Institutional Animal Care and Use Committee (IACUC No.: A-3158-01). The slicing solution consisted of the following (in mM): 85 NaCl, 75 sucrose, 24 NaHCO3, 25 glucose, 4 MgCl2, 2.5 KCl, 1.25 NaH2PO4, and 0.5 CaCl2. Slice storage and recording solutions (ACSF) were saturated with 95% O2/5% CO2 and consisted of the following (in mM): 119 NaCl, 26 NaHCO3, 11 glucose, 2.5 KCl, 2.5 CaCl2, 1.3 MgCl2, and 1 NaH2PO4. As in earlier studies, recordings were performed at room temperature (20–23°C) in order to limit GABA transporter activity and maximize tonic GABA current amplitude in perfused slices with no added GABA [18], [25]. For voltage-clamp recordings from cerebellar granule cells (holding potential of –70 mV), whole-cell pipettes contained the following (in mM): 140 CsCl, 10 HEPES, 1 EGTA, 4 Mg-ATP, and 0.4 GTP, titrated to pH 7.3 with CsOH. Voltage-clamp recordings of sEPSCs from cerebellar Golgi cells were obtained at a holding potential of –70 mV using pipettes containing (in mM): 126 K-gluconate, 4 KCl, 10 HEPES, 4 Mg-ATP, 0.3 Na-GTP and 10 phosphocreatinine. Loose-patch cell-attached (15–200 MΩ) recordings were obtained from Golgi cells which were visually identified by their location in the granule cell layer and characteristically large somata. Electrodes for loose-patch recordings contained the standard extracellular solution. Recordings were obtained in zero current mode and high-pass filtered at 1 Hz [26], [27]. Recording pipettes had a bath resistance of 5–10 MΩ. Recordings were performed using IR-DIC visualization techniques [25], [27] and obtained using Axon Instruments Axopatch 200B (Molecular Devices, Sunnyvale, CA). Tonic and synaptic GABA currents were recorded in perfusing ACSF containing no added GABA or GABA transporter blockers.

### Analysis and statistics

Whole-cell data were filtered at 5 kHz and acquired at a sampling rate of 20 kHz. Analysis was conducted using customized routines written in Igor Pro 7.0 (WaveMetrics, Lake Oswego, OR) [25], [28], [29]. IPSC τ_decay_ is reported as a weighted decay of bi-exponential fits to the average trace of over 10 events in each cell (Santhakumar et al.., 2006). Tonic GABAR-mediated current was defined as the steady-state current blocked by 10 µM gabazine (SR95513; [6-imino- 3-(4-methoxyphenyl)-1(6*H*)-pyridazinebutanoic acid hydrobromide]). The magnitude of tonic GABA currents was calculated by plotting all-point histograms of relevant 30 s segments of data. These data were fit to Gaussian equations, constraining fits to values two bins more negative than the peak. This ensured that the tail of higher-amplitude values (representing sIPSCs) did not influence the fit [25], [30]. In all cases, the effects of GABA agonists on tonic and synaptic GABA currents were compared with changes observed over otherwise identical sham perfusion periods. Events were visualized, and EPSCs with rapid kinetics as well as any 'noise' that spuriously met trigger specifications were rejected [25], [31]. Cumulative probability plots of frequency of individual sIPSCs were constructed using custom routines in IgorPro7.0, by pooling equal number of sIPSCs from each cell. The frequency of individual sIPSCs was calculated as the reciprocal of the inter-event interval between consecutive IPSCs. Statistical analysis was performed by paired and unpaired Student's *t*-test (Microsoft Excel 2007).Kolmogorov-Smirnov test (in IgorPro7.0), Wilcoxon Signed Rank Test or Mann-Whitney Rank Sum Test (Sigma Plot 12.3) were used, as appropriate, for data that failed the Shapiro-Wilk test for normality (Sigma Plot 12.3). One and two-way ANOVA and repeated measures ANOVA followed by post hoc pairwise comparisons were performed on Sigma Plot 12.3. Significance was set to *p* < 0.05. Since all recordings involved drug applications only one cell was recorded from each slice. Thus, data are reported from n cells were obtained from an identical number of slices. Data are shown as mean ± s.e.m or median and interquartile range (IQR) where appropriate.

## Results

**Figure 1:**
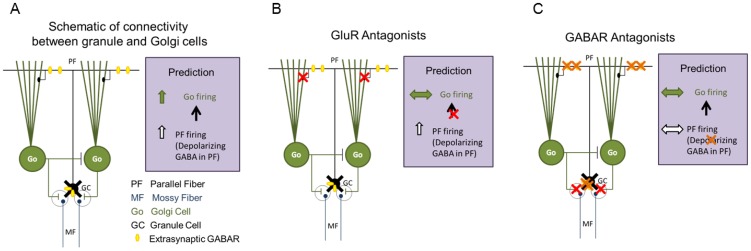
Schematic of the circuit hypothesis for ethanol-enhancement of synaptic GABA release from Golgi neurons. *A.* Schematic of the connectivity between cerebellar granule cells and Golgi cells including the inhibitory connections between Golgi cells identified in recent studies [33]. Location of extrasynaptic GABARs in granule cell somata and parallel fiber axons is illustrated. Prediction (right panel), illustrates the proposed changes in neuronal activity in the circuit following increased activation of parallel fiber GABARs. *B*. Hypothesized effect of GluR antagonists on circuit activity following increase in activation of granule cell extrasynaptic GABARs. Red X indicates blocked glutamatergic synapses. *C*. Predicted effects of GABAR antagonists on granule and Golgi cell activity following increased activation of granule cell extrasynaptic GABARs. Red X indicates blocked GABA synapses and orange X denotes block of both somato-dendritic and axonal extrasynaptic GABARs.

Previous work has shown that ethanol potentiates GABA release from cerebellar Golgi neurons. Changes in spontaneous, action potential-driven GABA release from Golgi neurons can be monitored by recording sIPSC frequency in cerebellar granule cells. Ethanol-induced increases in sIPSC frequency are prevented by tetrodotoxin [15] indicating that action potential firing, presumably in Golgi neurons, is required. Consistent with this finding, spiking activity in Golgi neurons increases in response to ethanol [15], [20], [21]. Apart from direct effects on Golgi cell membrane excitability [20], [21], ethanol may influence Golgi cell excitability by modulating activity levels in the cerebellar circuit. The latter possibility is supported by data demonstrating that low concentrations of ethanol enhance the inhibitory tone of granule cells which are the primary intrinsic source of glutamatergic input in the cerebellar circuit [18]. We set out to test the hypothesis that ethanol and other related modulators act indirectly to increase GABA release from cerebellar Golgi cells. This “circuit hypothesis” is based on the known anatomical connectivity of granule cells and Golgi cells within cerebellar cortex [32], [33], as illustrated in Figure 1. It posits that these drugs act on α6 and δ subunit-containing GABARs present only in cerebellar granule cells. Enhanced activity of extrasynaptic GABARs on granule cells is then hypothesized to indirectly excite Golgi cells (Fig. 1A). In the following sections, several predictions from this model are tested.

### Excitatory synaptic transmission is required for ethanol-induced increase in GABA release

**Figure 2:**
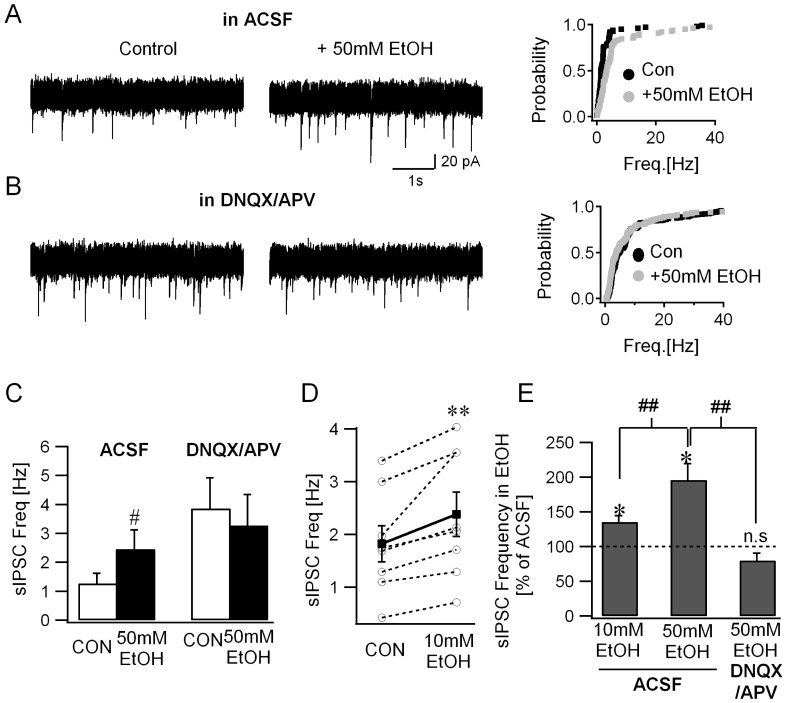
Blocking glutamatergic transmission abolishes ethanol-effects on granule cell sIPSC frequency. *A.* Representative granule cell current traces recorded in ACSF (CON, left panel) and during perfusion of 50 mM ethanol (EtOH, middle panel). Cumulative probability plots (right panel) compare the frequency of individual sIPSC in ACSF (CON) and after perfusion of 50 mM ethanol (same number of events from *n* = 5 cells; *p*<0.05 by K-S test). *B*. Granule cell current traces recorded in the presence of the GluR antagonists 20 µM APV and 25 µM DNQX (CON, left panel) and after inclusion of 50 mM ethanol (EtOH, middle panel). Cumulative probability plots (right panel) show the frequency distribution of individual sIPSCs in APV/DNQX (CON) and after addition of 50 mM ethanol (identical number of events from *n* = 6 cells; *p*<0.05 by K-S test). *C*. Summary data of the sIPSC frequency averaged over 30 second periods in control conditions before addition of ethanol (CON) and in the presence 50 mM ethanol. The frequency of sIPSCs recorded in ACSF is compared with the effect observed in DNQX/APV (# indicates p<0.05 by two-way repeated measures ANOVA followed by post-hoc analyses by Holm-Sidak method). *D*. Summary data of the sIPSC frequency averaged over 30 second periods in control conditions before addition of ethanol (CON) and in the presence 10 mM ethanol. Individual data points are represented by open circles connected by dotted lines. (** indicates p<0.05 by Wilcoxon Signed Rank test). *F*. Summary histogram of the sIPSC frequency in ethanol, normalized to frequency in the same cell prior to perfusion of ethanol (n.s. denotes p>0.05, * indicates p<0.05 by paired *t*-test and ## indicates p<0.05 by one-way ANOVA followed by post-hoc analyses by Holm-Sidak method).

The α6(R100Q) polymorphism has been shown to influence ethanol-modulation of tonic and synaptic GABA currents in cerebellar granule cells [18], [25]. This polymorphism is known to naturally occur in several rat strains including Sprague-Dawley rats, rat lines selectively bred for alcohol preference such as Sardinian rats, and rats selectively bread for alcohol sensitivity [18], [34]–[36]. While both alcohol-tolerant rats homozygous for α6^100R^ and alcohol-non-tolerant rats homozygous for α6^100Q^ demonstrate modulation of tonic GABA currents by ethanol, the magnitude of the alcohol induced enhancement of tonic GABA currents was shown to be greater in rats homozygous for α6^100Q^ allele [18]. To eliminate potential confounding effects of differences in genotype and to maximize ethanol modulation of cerebellar granule cell tonic GABA currents all rats used in the current study were homozygous for α6^100Q^. As reported previously, ethanol (50 mM) significantly enhanced the frequency of spontaneous inhibitory synaptic currents (sIPSCs) in cerebellar granule cells (Fig 2A). Cumulative probability distributions of the instantaneous frequency of granule cell sIPSCs showed a statistically significant increase in the presence of 50 mM ethanol (Fig 2A-panel to the right. sIPSC frequency in Hz, ACSF: median = 1.02, IQR = 1.47–2.44, n = 5 cells from 4 rats; 50 mM EtOH: median = 1.90, IQR = 3.45–5.06, n = 5 cells from 4 rats, p<0.05 K-S test). Granule cell sIPSC frequency, measured as the averaged frequency over a 30 sec recording period, was also enhanced in 50 mM ethanol (Fig. 2C; sIPSC frequency in Hz, ACSF: 1.27±0.35; 50 mM EtOH: 2.46±0.66, n = 5 cells from 4 rats, p<0.05 paired *t*-test; effect of 50 mM EtOH showed partial recovery on washout, wash: 1.89±0.75). These data are consistent with previous studies identifying ethanol-induced increases in granule cell sIPSC frequency [15], [18]. Since α6^100Q/100Q^ rats show enhanced ethanol-sensitivity [18], we conducted an additional set of experiments to examine whether 10mM ethanol, a concentration under the legal limit for motor vehicle operation, enhances synaptic inhibition in granule cells. Consistent with the effects at higher concentrations, granule cell sIPSC frequency was also increased by 10 mM ethanol (Fig. 2D; sIPSC frequency in Hz, ACSF: 1.82±0.34; median = 1.71, IQR = 1.15–2.74, 10 mM EtOH: 2.38±0.42, median = 2.10, IQR = 1.40–3.55, an increase in sIPSC frequency in all 8 cells tested from 3 rats, p = 0.008 by Wilcoxon Signed Rank test, a 135.31±8.87% increase in sIPSC frequency in 10 mM EtOH compared to the frequency in ACSF). Expressed as a fractional increase in individual cells, ethanol resulted in dose-dependent enhancement of sIPSC frequency to 135.31±8.87% in 10 mM EtOH and 196.22±23.24% in 50 mM EtOH when normalized to the frequency in ACSF (Fig. 2E; p<0.05, One-way ANOVA followed by Holm-Sidak method for pairwise comparison).

Having established consistent and dose-dependent ethanol-induced increases in granule cell sIPSC frequency in ACSF, we next tested whether ethanol-enhancement of synaptic release was preserved when granule cells were synaptically uncoupled from the cerebellar network (Schematic in Fig. 1B). As illustrated in Fig 2B, ethanol (50 mM) failed to enhance granule cell sIPSC frequency in the presence of NMDA and AMPA/Kainate receptor antagonists, APV (20 µM) and DNQX (25 µM) respectively. Notably, the cumulative probability distribution of granule cell instantaneous sIPSCs frequencies showed a small decrease in 50 mM ethanol (Fig 2B-panel to the right. sIPSC frequency in Hz, APV/DNQX: median = 2.41, IQR = 3.86–9.03, n = 6 cells from 4 rats; APV/DNQX+50 mM EtOH: median = 1.99, IQR = 3.86–9.03, n = 6 cells, p<0.05 K-S test). This contrasts with the expected increase in frequency if ethanol actions were entirely due to modulation of intrinsic excitability of Golgi cells. Correspondingly, there was no increase in granule cell sIPSC frequency, averaged over a 30 sec recording period (Fig. 2C; sIPSC frequency in Hz, APV+DNQX: 3.87±1.06; +50 mM EtOH: 2.27±1.07, n = 6 cells from 4 rats, p>0.05 paired *t*-test). Two-way repeated measures ANOVA for interaction between the effect of 50 mM EtOH and APV+DNQX on sIPSC frequency revealed a significant interaction between 50 mM EtOH and APV+DNQX (F(1,21) = 10.51, p<0.05). Subsequent post hoc analyses by Holm-Sidak method for pairwise comparison identified a significant effect for 50 mM EtOH in control ACSF (sIPSC frequency in control ACSF in Hz, ACSF: 1.27±0.35; 50 mM EtOH: 2.46±0.66, n = 5 cells from 4 rats, p<0.05) while 50 mM EtOH failed to modulate sIPSC frequency in APV+DNQX (APV+DNQX: 3.87±1.06; +50 mM EtOH: 2.27±1.07, n = 6 cells from 4 rats, p>0.05). Additionally, sIPSC frequency in ethanol, normalized to the frequency prior to ethanol perfusion was not increased in the presence of glutamate receptor (GluR) antagonists (Fig. 2E; 79.88±9.44% p>0.05 by *t*-test). Comparison of sIPSC frequency following perfusion of ethanol, normalized to the frequency prior to ethanol in experiments conducted in ACSF or in APV+DNQX identified a significant difference between groups (Fig. 2E; 10 mM EtOH in ACSF: 135.31±8.87%, 50 mM EtOH in ACSF: 196.22±23.24%, 50 mM EtOH in APV+DNQX: 79.88±9.44%, F(2, 18) = 16.08, p<0.05 by one-way ANOVA with p<0.05 for pairwise comparisons of 10 mM EtOH in ACSF vs. 50 mM EtOH in ACSF, and 50 mM EtOH in ACSF vs. 50 mM EtOH in APV+DNQX by post hoc analyses using the Holm-Sidak method).

**Figure 3:**
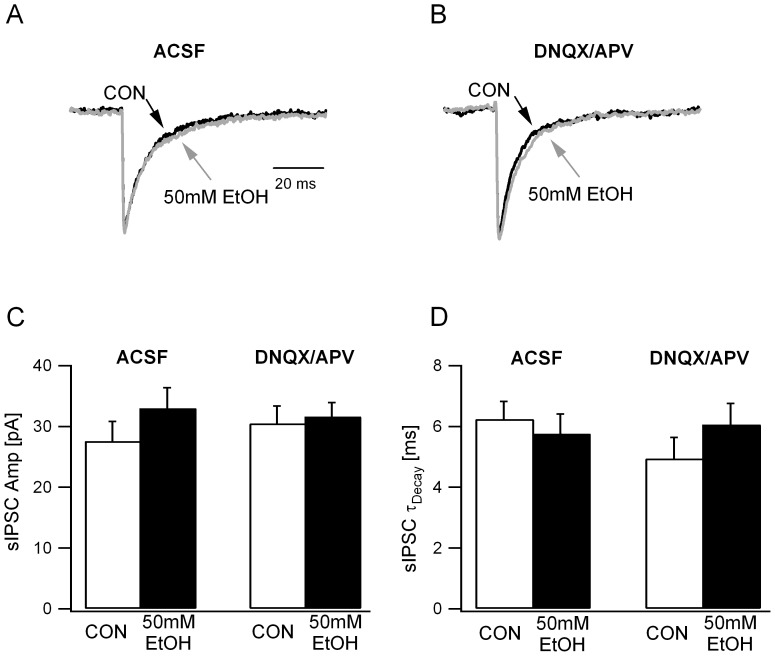
Ethanol does not modulate sIPSC amplitude or decay. *A.* Overlay of normalized granule cell sIPSC traces recorded in ACSF show that there was no difference in the IPSC kinetics under control conditions (black) and in 50mM ethanol (gray). *B.* Overlay of normalized granule cell sIPSC traces recorded in DNQX (25 µM) and APV (20 µM) show no difference in IPSC kinetics in the absence (black) and in 50mM ethanol (gray). *C*. Summary plots of sIPSC amplitude before and during ethanol perfusion illustrate the lack of ethanol-modulation both in ACSF and in GluR antagonists (DNQX/APV). *D*. Histogram of the weighted τ_decay_ of granule cell sIPSCs in ACSF in GluR antagonists (DNQX/APV) recorded before and during ethanol perfusion.

In contrast to the differential effects of ethanol on sIPSC frequency in ACSF and GluR antagonists, mean sIPSC peak amplitude was not modulated by ethanol in either condition (Fig. 3C; sIPSC amplitude in pA, ACSF: 27.59±3.23; +50 mM EtOH: 32.96±4.1, n = 6 from 4 rats,; APV+DNQX: 30.49±2.88; +50 mM EtOH: 31.63±1.80, n = 6 cells from 4 rats, F(1,15) = 1.6, p>0.05 for effect of 50 mM EtOH, F(1,15) = 0.25, p>0.05 for effect of APV+DNQX and F(1,15) = 1.99, p>0.05 for interaction between EtOH and APV+DNQX by two-way repeated measures ANOVA). Similarly, ethanol failed to alter sIPSC decay time-constant both in ACSF and in the presence of GluR antagonists (Fig. 3A-B & D; sIPSC τ_decay_ ms, ACSF: 6.25±0.59; +50 mM EtOH: 5.76±0.49, n = 6 from 4 rats; APV+DNQX: 4.95±0.69; +50 mM EtOH: 6.07±0.7, n = 6 cells from 4 rats, F(1,15) = 1.6, p>0.05 for effect of 50 mM EtOH, F(1,15) = 0.25, p>0.05 for effect of APV+DNQX and F(1,15) = 1.99, p>0.05 for interaction between EtOH and APV+DNQX by two-way repeated measures ANOVA). These data indicate that circuit mechanisms involving excitatory synaptic transmission mediate ethanol-enhancement of granule cell synaptic inhibition.

**Figure 4:**
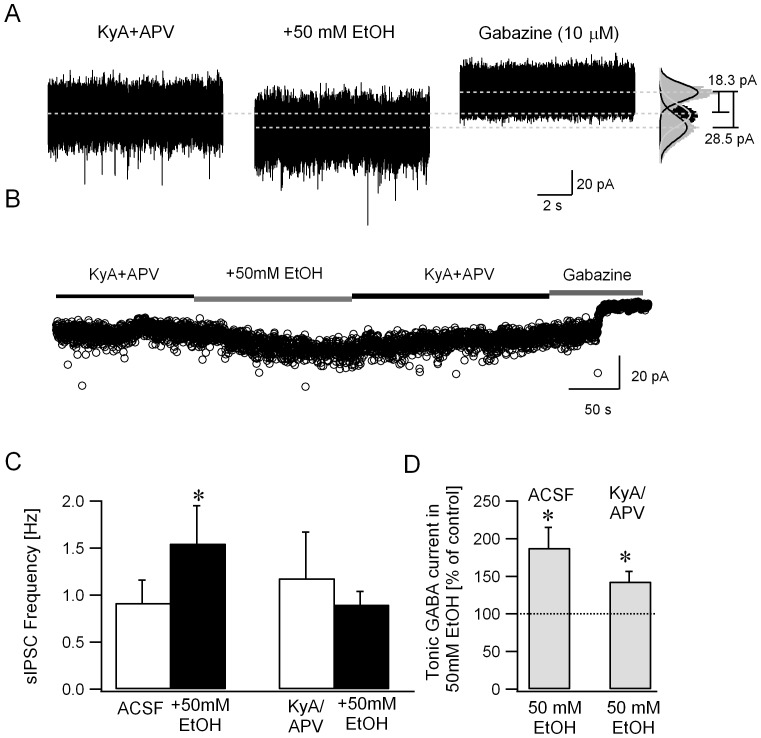
Ethanol enhances tonic GABA currents without increasing sIPSC frequency in the presence of glutamate receptor antagonists. *A*. Voltage clamp recordings from a granule cell illustrates the effect of ethanol (50 mM) on synaptic and tonic GABA currents recorded in the presence of the GluR antagonists APV (20 µM) and kynurenic acid (KyA, 3 mM). To the right are histograms of all points in each segment. Tonic GABA currents were measured as the baseline current blocked by GABAR antagonist gabazine (10 µM). Gaussian fits to the positive half of the current trace under each condition are superimposed (right panel). The dashed lines indicate the mean current from these fits and difference currents are noted. *B*. Plot illustrates the time course of changes in baseline currents, averaged over 100 ms time intervals, during perfusion of ethanol (50 mM) and subsequent washout of ethanol. Baseline current averages during gabazine perfusion at the end of the recordings were used to measure tonic GABA current amplitude. Segments of representative current traces in A and baseline currents in B were obtained from the same recordings *C.* Summary plot of the effect of ethanol on granule cell sIPSC frequency in the presence of ACSF and in KyA/APV. In each case, sIPSC frequency was averaged over a 30 second recording periods. *C*. Histogram shows that the amplitude of tonic GABA currents in ethanol (50 mM) normalized to the tonic GABA current amplitude in the same cell prior to perfusion of ethanol, both in ACSF and in KyA/APV. * indicates p<0.05 by paired *t*-test.

A potential confound exists in the experiments described above in that granule cell sIPSC frequency was increased by APV and DNQX prior to application of ethanol (sIPSC frequency in Hz, ACSF: 1.25±0.20, n = 11 cells from 7 rats; APV+DNQX: 3.87±1.06, n = 6 cells from 4 rats, p<0.05 *t*-test). Similarly, APV and DNQX increased the amplitude of tonic GABA currents all cells tested, although the increase failed to reach statistical significance (tonic GABA currents in pA, ACSF: 11.3±4.1; APV+DNQX: 24.1±15.1, n = 4 cells from 3 rats, p>0.05 by *t*-test). Indeed, it is well known that quinoxalinedione derivatives such as CNQX and DNQX can increase inhibitory neuronal firing due to their actions as partial agonists at certain GluR subtypes [37]–[39]. In order to rule out potentially confounding ceiling effects, we examined ethanol (50 mM) modulation of sIPSC frequency in kynurenic acid (KyA, 3 mM) and APV (20 µM), a GluR antagonist cocktail which does not increase Golgi cell firing [37], [39]. Granule cell sIPSC frequency in APV and KyA was not different from the frequency in ACSF (sIPSC frequency in Hz, ACSF: 1.25±0.20, n = 11 cells from 7 rats; APV+KyA: 1.18±0.49, n = 8 cells from 3 rats, p>0.05 *t*-test), confirming the absence of non-specific increase in sIPSC frequency in KyA. Importantly, ethanol (50 mM) increased granule cell sIPSC frequency in ACSF but failed to do so in the presence of KyA and APV (Fig. 4A, C, sIPSC frequency in Hz, ACSF: 0.92±0.24, ACSF+EtOH: 1.55±0.40, n = 6 cells from 4 rats, p = 0.036 paired *t*-test; APV+KyA: 1.18±0.49, APV+KyA+EtOH: 0.90±0.15, n = 8 cells from 5 rats, p = 0.94 by paired *t*-test). Similar to the findings in APV and DNQX, sIPSC peak amplitude and decay time-constant were not modulated by ethanol (data not shown). These data demonstrate that granule cell output plays a crucial role in ethanol-enhancement of the frequency of granule cell synaptic inhibitory inputs.

### Ethanol-modulation of granule cell tonic GABA currents does not require increases in presynaptic GABA release

As reasoned earlier, the circuit hypothesis of ethanol action is based on results of earlier studies demonstrating that ethanol augments currents in extrasynaptic GABAR isoforms in recombinant systems [18], [40]–[43] and tonic GABA currents in cerebellar granule cells [15], [18]. A critical controversy concerning the mechanism of ethanol action is whether ethanol-enhancement of granule cell tonic GABA currents results from direct ethanol action on extrasynaptic GABA receptors or a consequence of increases in synaptic GABA release by Golgi cells [1], [44], [45]. Our experiments in APV and KyA are ideally suited to address this issue. If, as we propose, ethanol directly enhances extrasynaptic GABARs, ethanol-induced increase in sIPSC but not tonic GABA currents will be abolished by GluR antagonists. If, on the other hand, ethanol augments granule cell tonic GABA currents secondary to increases in synaptic inhibition, elimination of ethanol-enhancement of sIPSC frequency in GluR antagonists (Fig 4A, C) would preclude an increase in tonic GABA currents. To test this, we measured whether ethanol effects on granule cell tonic GABA currents persisted in the presence of APV and KyA. As illustrated by a plot showing the baseline currents averaged over 100 ms time intervals (Fig. 4B), ethanol (50 mM) reversibly increased the baseline tonic GABA currents in APV and KyA. Under these conditions ethanol (50 mM) increased granule cell tonic GABA current amplitude to 143.18±12.67% (from 13.6±2.5 pA in APV+KyA to 18.1±2.6 pA in EtOH+APV+KyA, n = 8 cells from 5 rats, p<0.05 by paired *t*-test) in the presence of GluR antagonists (Fig. 4D). Although the average magnitude of ethanol-enhancement of tonic GABA currents in GluR antagonists was lower than that observed in ACSF (EtOH enhancement of tonic GABA currents: 187.9±27.1% p<0.05 by *t*-test from 10.4±3.0 pA in ACSF to 17.2±4.7pA in EtOH+ACSF, n = 6 cells from 4 rats in ACSF and 143.18±12.67% in APV+KyA, p>0.05 by *t*-test; tonic GABA currents 17.2±4.7pA in EtOH+ACSF, n = 6 cells from 4 rats and 18.1±2.6 pA in EtOH+APV+KyA, n = 8 cells from 5 rats, p>0.05), the difference did not reach statistical significance (EtOH enhancement of tonic GABA currents: 187.9±27.1% median = 162.36, IQR = 109.36–260.03 in n = 6 cells from 4 rats in ACSF ACSF and 143.18±12.67% median = 143.59, IQR = 105.07–181.15 in n = 8 cells from 5 rats APV+KyA, p = 0.66 by Mann-Whitney Rank Sum Test). It is possible that the reduction in ethanol-potentiation of tonic GABA currents in GluR antagonists reflects the lack of network-based increases in synaptic GABA release. However, our data demonstrate that ethanol can enhance granule cell tonic GABA currents even in the absence of increases in synaptic GABA release and show that ethanol-induced increase in synaptic GABA release requires a functional glutamatergic circuit.

### Agonists selective for extrasynaptic GABA receptors enhance inhibitory synaptic inputs to granule cells

**Figure 5:**
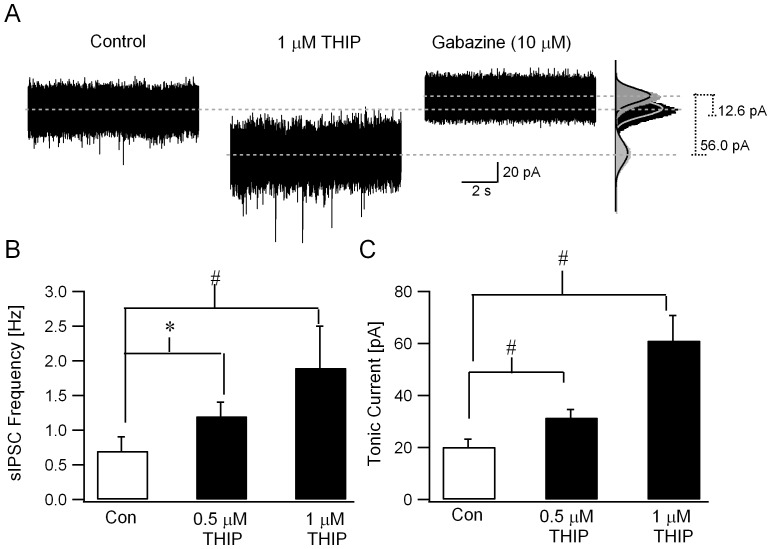
GABAR δ-subunit selective agonist THIP increases sIPSC frequency. *A.* Representative current traces illustrate enhancement of sIPSC frequency and tonic GABA currents by THIP (1 µM). To the right are histograms of all points in each segment. Tonic GABA currents were measured as the baseline current blocked by GABAR antagonist gabazine (10 µM). Gaussian fits to current trace under each condition are superimposed (right panel). Dashed lines indicate the mean baseline currents and the difference in baseline currents measured as tonic GABA currents are noted. *B.* Summary data show the effect of 500 nM and 1 µM THIP on granule cell sIPSC frequency. *C.* Histogram shows the amplitude of tonic GABA currents in ACSF, 500 nM and 1 µM THIP measured simultaneously during the recordings in B. * indicates p<0.05 by paired *t*-test, # indicates p<0.05 by one-way repeated measures ANOVA followed by post-hoc analyses by Holm-Sidak method.

If modulation of granule cell extrasynaptic GABARs underlie the effects of ethanol on synaptic inhibition, one would expect that, in addition to ethanol, compounds that selectively potentiate extrasynaptic GABARs would cause similar increases in the frequency of synaptic inhibition. We examined whether THIP (4,5,6,7-tetrahydroisoxazolo(5,4-c)pyridin-3(-ol) or gaboxadol) [46], a highly potent and selective agonist of extrasynaptic GABA receptors at submicromolar concentrations [10], [11], augments granule cell sIPSC frequency. Consistent with effects on extrasynaptic GABARs, THIP (500 nM) increased tonic GABA currents from 20.2±3.0 pA in ACSF to 31.6±3.1 pA (Fig. 5C, n = 5 cells from 3 rats, p< 0.05, paired *t*-test). In parallel, THIP enhanced the frequency of granule cell sIPSC from 0.53±0.15 Hz in ACSF to 1.17±0.20 Hz (Fig. 5B, n = 5 cells from 3 rats, p< 0.05, paired *t*-test). Thus, THIP simultaneously increased both the magnitude of tonic GABA currents and the frequency of sIPSCs in cerebellar granule cells, as predicted on the basis of the circuit hypothesis. Increases in sIPSC frequency by THIP were observed in spite of a small decrease in sIPSC amplitude, which might have lowered the detection of sIPSCs. The decrease in amplitude in 500 nM THIP, however, was not statistically significant (in pA, ACSF: 23.46±2.00; THIP: 19.72±2.15, n = 5 cells from 3 rats, p>0.05, paired *t*-test). At higher concentrations, THIP (1 µM) caused a significant decrease in sIPSC amplitude (in pA, ACSF: 23.92±2.21; THIP: 14.67±1.80, n = 5 cells from 3 rats, p<0.05, paired *t*-test). However, THIP (1 µM) still augmented sIPSC frequency (Fig. 5A,B, in Hz, ACSF: 0.77±0.23; THIP: 1.93±0.20, n = 5 cells from 3 rats, (F2,15) = 8.96, p< 0.05 by one-way repeated measures ANOVA, p<0.05 for effect of 1µM THIP on sIPSC frequency by post hoc comparison using Holm-Sidak method) and tonic GABA current amplitude (Fig. 5A,C, in pA, ACSF: 20.2±3.0; THIP: 63.7±5.3, n = 5 cells from 3 rats, (F2,19) = 15.4, p< 0.05 by one-way repeated measures ANOVA, p<0.05 for effect of 1 µM THIP on tonic GABA current amplitude by post hoc comparison using Holm-Sidak method). The difference in sIPSC frequency between 500 nM THIP and 1 µM THIP did not reach statistical significance (Fig. 5B, in Hz, 500 nM THIP F: 1.17±0.20; 1 µM THIP: 1.93±0.20, n = 5 cells from 3 rats, p>0.05 by post hoc comparison using Holm-Sidak method following one-way repeated measures ANOVA).

**Figure 6:**
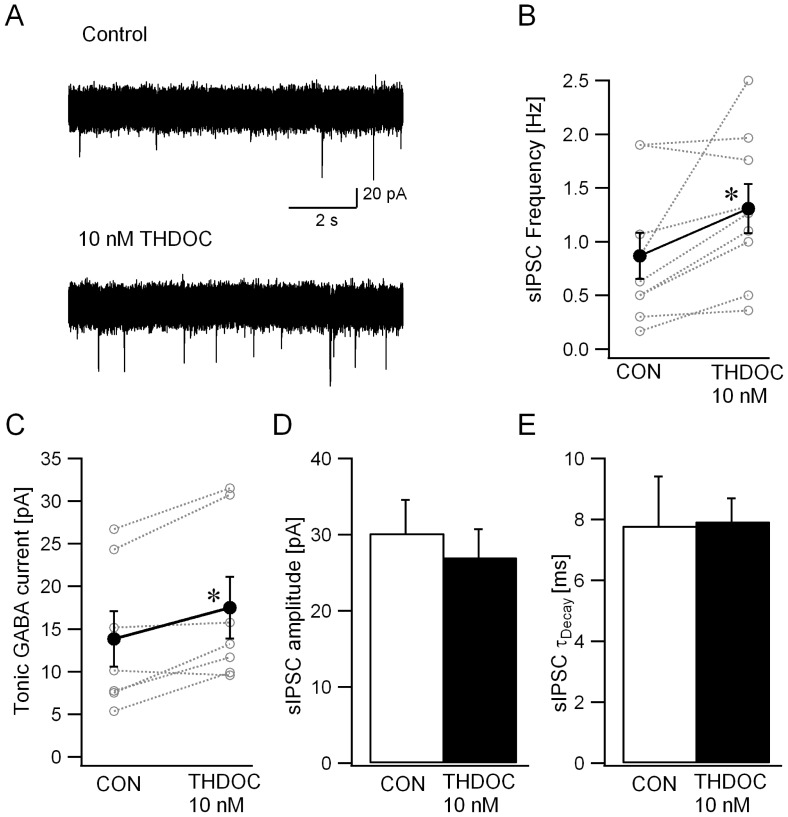
Neurosteroids induce parallel increases in granule cell sIPSC frequency and tonic GABA currents. A. Voltage clamp recordings from granule cells illustrate the relatively low frequency of synaptic events in granule cells recorded in control conditions (upper trace) and the increase in the number of synaptic events following perfusion of 10 nM THDOC (lower trace). *B-C*. Plots show the effect of THDOC (10 nM) on the frequency of sIPSCs (*B*) and amplitude of tonic GABA current (*C*) recorded in the same cells. Individual data points are represented by open circles connected by dotted lines. Synaptic events were completely abolished in 10 µM gabazine. Amplitude of tonic GABA currents was measured as the baseline current blocked by 10 µM gabazine. * indicates p<0.05 by paired *t*-test, *D-E*. Histogram demonstrates the lack of THDOC (10 nM) modulation of granule cell sIPSC amplitude (*B*) and weighted τ_decay_ (*D*).

To provide further evidence that the above effects are a general feature of extrasynaptic GABAR agonists, we tested the neurosteroid THDOC, which, at a concentration of 10 nM, selectively increases tonic GABA currents without modulating sIPSC amplitude and kinetics in dentate and cerebellar granule cells [5]. Like ethanol and THIP, THDOC (10 nM) increased granule cell sIPSC frequency (Fig. 6A&B, in Hz, ACSF: 0.87±0.21; THDOC: 1.31±0.23, n = 9 cells in 9 rats, p = 0.03, paired *t*-test). In the same recordings, tonic GABA currents were increased from 13.8±3.2 pA in ACSF to 17.5±3.6 pA in THDOC (Fig. 6C, n = 9 cells from 9 rats, p = 0.01, paired *t*-test). Consistent with earlier studies [5], sIPSC amplitude and decay were not altered by THDOC (Fig. 6D&E, sIPSC amplitude in pA, ACSF: 30.2±4.38; THDOC: 27.03±3.67, n = 9 cells from 9 rats, p = 0.52, paired *t*-test; sIPSC τ_decay_ in ms, ACSF: 7.79±1.61; THDOC: 7.93±0.75, n = 9 cells from 9 rats, p = 0.93, paired *t*-test). Together, these data demonstrate that three pharmacologically distinct compounds, including two agents that selectively modulate extrasynaptic GABARs, caused similar increases in granule cell sIPSC frequency. These findings, together with the block of ethanol-enhancement of sIPSC frequency in GluR antagonists, suggest that circuit-based mechanisms augment presynaptic GABA release following enhancement of extrasynaptic GABARs in granule cells. In addition to increasing synaptic inhibition, such a circuit driven potentiation of GABA release would elevate ambient GABA levels and reinforce increases in tonic GABA currents.

### Contribution of GABA and glutamate receptors to enhancement of Golgi cell activity by extrasynaptic GABAR agonists

**Figure 7:**
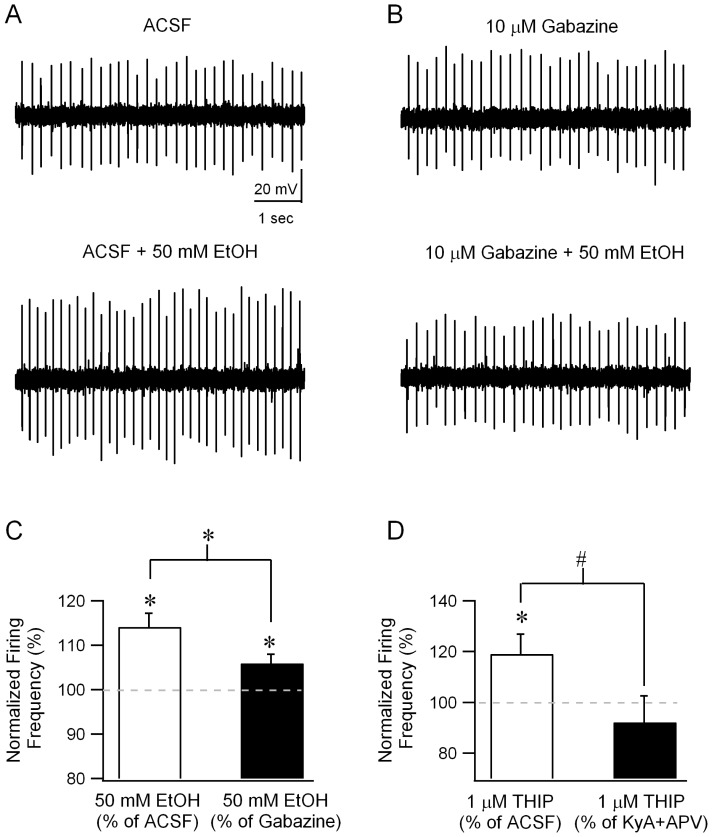
Blocking GABA or glutamate receptors reduces enhancement of Golgi cell firing by extrasynaptic GABAR agonists. *A.* Example trace of loose-patch cell-attached recordings from a Golgi cell illustrates the spontaneous firing in ACSF (upper panel) and the increase in firing in the presence of 50 mM ethanol (lower panel). *B.* Golgi cell firing recorded in the presence of gabazine (10 µM) in loose-patch mode before (upper panel) and during application of 50 mM ethanol (lower panel). *C*. Summary plots of the frequency of Golgi cell firing in 50 mM ethanol, normalized to the firing frequency before ethanol perfusion in ACSF (white bar) and in gabazine (black bar). *D*. Summary plots of the frequency of Golgi cell firing in 1 µM THIP, normalized to the firing frequency before THIP perfusion in ACSF (white bar) and in 3 mM kynurenic acid and 20 µM APV (black bar). * indicates p<0.05 by paired *t*-test.

In the experiments detailed above, we demonstrate that modulating extrasynaptic GABARs in granule cells influences sIPSC frequency. Since Golgi cells are the principle source of synaptic inhibition to granule cells [47], [48], increases in Golgi cell activity must underlie the enhanced granule cell sIPSC frequency. Indeed, earlier studies have shown that Golgi cell firing is increased by ethanol [15]. If ethanol acts by way of increasing granule cell GABA currents, GABAR antagonists would be expected to reduce or eliminate the enhancement of Golgi cell firing by ethanol (Fig. 1C). To directly test the involvement of GABARs, we compared ethanol-modulation of Golgi cell firing in cell-attached recordings performed in the absence and presence of gabazine (10 µM). As reported previously [15], under control conditions, bath application of ethanol (50 mM) increased Golgi cell firing to 114.11±3.03% of the frequency in ACSF (Fig. 7A&C; frequency in Hz, ACSF: 7.11±1.28; +EtOH: 7.98±1.28, n = 6 cells from 3 rats, p<0.05 by paired *t*-test). Switching the perfusion from control ACSF to gabazine (10 µM) resulted in a small increase in Golgi cell firing which did not reach statistical significance (frequency in Hz, ACSF: 7.11±1.28, median = 6.12, IQR = 4.68–10.78; Gabazine: 7.34±1.17, median = 7.42, IQR = 4.58-10.28, n = 6 p>0.05 by Wilcoxon Signed Rank Test). Crucially, in the presence of gabazine (10 µM), ethanol-enhancement of Golgi cell firing was significantly reduced (Fig. 7B&C frequency in EtOH normalized to frequency before EtOH, in ACSF: 114.11±3.03%, in gabazine: 105.96±2.02%, n = 6 cells from 3 rats, p<0.05 by paired *t-*test). Since Golgi cells are not known to express extrasynaptically located GABARs, the observed decrease in ethanol-modulation of Golgi cell firing in the presence of GABAR antagonists indicates that changes in synaptic inputs contribute ethanol-enhancement of Golgi cell firing. However, ethanol did increase Golgi cell firing frequency in the presence of gabazine (Fig. 7C; frequency in Hz, Gabazine: 7.34±1.17; +EtOH: 7.76±1.26, n = 6 cells from 3 rats, p<0.05 by paired *t*-test) indicating that ethanol can also directly increase Golgi cell firing [20]. These experiments demonstrate that although ethanol contributes to direct enhancement of Golgi cell firing in the presence of GABA receptor antagonists, in the intact cerebellar circuit, modulation of GABAergic inhibition contributes to robust circuit-based enhancement of Golgi cell activity by ethanol.

To further test whether ethanol modulation of tonic GABA currents is shared by selective modulators of extrasynaptic GABA currents, we examined the effect of THIP (1 µM) on Golgi cell firing. In cell attached recordings from Golgi cells, bath application of THIP (1 µM) enhanced Golgi cell firing frequency (Fig. 7D, frequency in THIP when normalized to the frequency in ACSF: 119.13±7.75%, n = 9 cells from 5 rats, p<0.05 by paired *t-*test). Next we verified the contribution of glutamatergic inputs to THIP-induced enhancement of Golgi cell firing frequency by testing the effect of THIP (1 µM) in the presence of glutamate receptor antagonists (3 mM kynurenic acid and 20 µM APV). In contrast to its effect in ACSF, THIP failed to modulate Golgi cell firing frequency in the presence of APV and kynurenic acid (Fig. 7D, frequency in THIP normalized to the frequency in KyA and APV: 92.2±8.65%, n = 9 cells from 5 rats, p>0.05 by paired *t-*test). The effect of glutamate receptor antagonists on THIP-modulation of Golgi cell firing was statistically significant (F (1, 35)  =  4.32, p< 0.05 by two-way repeated measures ANOVA followed by post hoc comparison using Holm-Sidak method).

**Figure 8:**
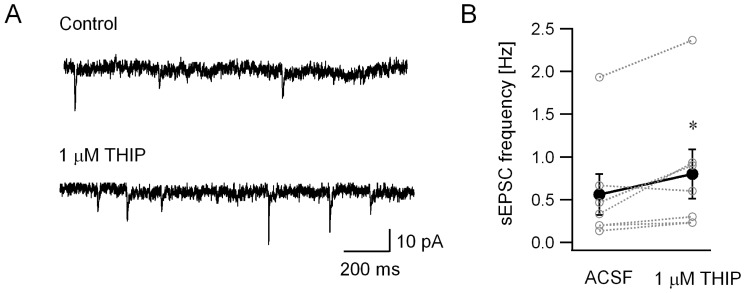
THIP increases Golgi cell sEPSC frequency. *A.* Representative Golgi cell current recordings obtained with a low chloride internal solution at a holding potential of –70 mV. Recordings in control ACSF (upper panel) and after perfusion of 1 µM THIP (lower panel) illustrate the increase in frequency of spontaneous excitatory synaptic events in THIP. *B*. Summary plot shows the effect of THIP on Golgi cell sEPSC frequency. Individual data points are represented by open circles connected by dotted lines. * indicates p<0.05 by paired *t*-test.

To confirm that agonists of extrasynaptic GABA receptors modulate glutamatergic inputs to Golgi cells, we directly assessed the effect of THIP on excitatory synaptic inputs to Golgi cells. In whole cell, voltage clamp recordings obtained using a low chloride internal solution, THIP (1 µM) enhanced the frequency of spontaneous excitatory postsynaptic currents (sEPSCs) in Golgi cells (Fig. 8A&B, sEPSC frequency in Hz, ACSF: 0.56±0.24 Hz; THIP: 0.80±0.29 Hz, n = 7 cells from 3 rats, p<0.05 by paired *t*-test). However, THIP failed to alter sEPSC charge transfer (sEPSC charge transfer in pA*ms = fC, ACSF: 79.82±9.28; THIP: 77.7±12.73, n = 7 cells from 3 rats, p>0.05 by paired *t*-test). Consistent with the proposed circuit-based mechanism, these experiments demonstrate that increases in spontaneous excitatory synaptic inputs underlie enhancement of Golgi cell firing by THIP, an agonist selective for extrasynaptic GABA receptors at concentrations used in this study.

### Ethanol-potentiation of presynaptic GABA release requires the presence of extrasynaptic GABARs in postsynaptic granule cells

**Figure 9:**
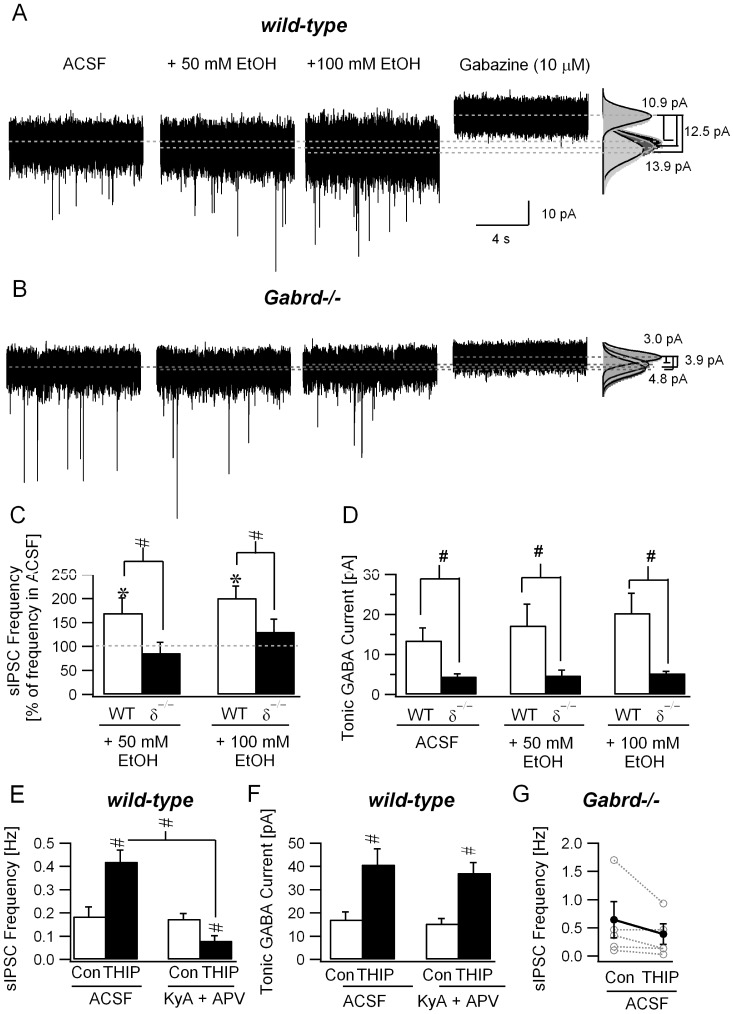
Increase in granule cell sIPSC frequency by ethanol and THIP require the presence of GABAR δ–subunits and intact glutamatergic synaptic transmission. *A-B.* Current traces recorded in cerebellar granule cells from wild-type (*A*) and *Gabrd*–*/*– mice (*B*) in the presence of 50mM and 100 mM ethanol. Both synaptic and tonic GABA currents are blocked by gabazine (10 µM). Gaussian fits to current traces superimposed on histograms of all points in each segment (right panel). Dashed lines indicate the mean current from the fits used to measure tonic GABA current amplitude (difference in baseline current blocked by gabazine). *C*. sIPSC frequency in ethanol normalized to frequency prior to ethanol perfusion in wild-type (WT) and *Gabrd*–*/*– mice (δ^−/−^). *D*. Granule cell tonic GABA current amplitudes in wild-type (WT) and *Gabrd*–*/*– (δ^−/−^) measured in control ACSF and in the presence of 50 and 100 mM ethanol. *E*. Summary plot of the effect of THIP (500 nM) on granule cell sIPSC frequency in wild-type (WT) mice, in ACSF and in the presence of KyA/APV. In each case, sIPSC frequency was averaged over a 30 second recording period. *F*. Effect of THIP (500 nM) on granule cell tonic GABA current amplitude in wild-type (WT) mice measured in control ACSF and in the presence of KyA/APV. * indicates p<0.05 by paired *t*-test within genotype and # indicates p<0.05 by two-way repeated measures ANOVA followed by post hoc pairwise comparison using Holm-Sidak method. *G*. Effect of THIP (1 µM) on granule cell tonic GABA current amplitude in *Gabrd*–*/*– mice measured in control ACSF. Individual data points are represented by open circles connected by dotted lines.

We used *Gabrd*–/– mice to directly examine whether modulation of extrasynaptic GABARs is necessary for ethanol-enhancement of granule cell sIPSC frequency. As observed in rats, 50 mM ethanol enhanced sIPSC frequency (Fig. 9A&C; in Hz, ACSF: 0.35±0.08; EtOH: 0.45±0.11, n = 6 cells from 5 mice, p<0.05 by paired *t-*test; frequency in 50 mM EtOH normalized to ACSF 170.10±24.91%, p<0.05 by *t-*test) and enhanced tonic GABA currents (Fig. 9A&D; in pA, ACSF: 13.5±3.1; EtOH: 17.3±5.3 n = 6 cells from 5 mice, p<0.05 by paired *t-*test, tonic GABA currents in EtOH normalized to ACSF 124.26±6.33%, p<0.05 by *t-*test) in cerebellar granule cells from wild-type mice. Tonic GABA currents in control ACSF were substantially reduced in *Gabrd*-/- mice (Fig. 9B&D; in pA, WT: 13.5±3.1, n = 6 cells from 3 mice; *Gabrd*–*/*–: 4.5±0.6, n = 6 cells from 3 mice, p<0.05 by *t*-test) as has been previously reported [5]. In contrast to the effect in wild-type mice, ethanol (50 mM) failed to increase granule cell sIPSC frequency in *Gabrd*–*/*– mice (Fig. 9B&C; sIPSC frequency in Hz, ACSF: 0.61±0.20; EtOH: 0.65±0.29, n = 7 cells from 3 mice, frequency in 50 mM EtOH normalized to ACSF 86.48±22.37%, p>0.05 by paired t-test). Moreover, as expected based on the lack of GABAR δ subunits, the residual tonic GABA currents in *Gabrd*–*/*– mice was not modulated by ethanol (Fig. 9B&D; tonic GABA current in pA, ACSF: 4.5±0.6; EtOH: 4.8±1.2, tonic GABA currents in EtOH normalized to ACSF 103.85±15.05%, p>0.05 by *t*-test). Ethanol, even at a concentration of 100 mM did not lead to substantive increases in either sIPSC frequency (Fig. 9B&C; sIPSC frequency in Hz, ACSF: 0.61±0.20; 100 mM EtOH: 0.70±0.27, n = 6 cells from 3 mice, p>0.05 by paired *t*-test) or tonic GABA currents (Fig. 9B&D; tonic GABA current in pA, ACSF: 4.5±0.6; 100 mM EtOH: 5.3±1.3, n = 6 cells from 3 mice, p>0.05 by *t*-test) in *Gabrd*–*/*– mice. Summary data demonstrate that enhancement of granule cell sIPSC frequency by 50 and 100 mM ethanol was abolished in granule cells from *Gabrd*–*/*– mice (Fig. 9C), in which residual tonic GABA currents lacked ethanol-modulation (effect of genotype was significant F (1, 30)  = 7.34, p<0.05 by two-way repeated measures ANOVA followed by post hoc test by Holm-Sidak method).

Finally, we examined whether low concentrations of THIP (500 nM) can modulate sIPSC frequency in granule cells from WT mice. As illustrated by the summary data in Fig 9E, THIP (500 nM) increased sIPSC frequency in granule cells in control ACSF (sIPSC frequency in Hz, ACSF: 0.19±0.04; 500 nM THIP 0.42±0.05, n = 6 cells from 3 mice, p<0.05 by paired *t*-test). However, as would be expected based on a circuit effect, THIP (500 nM) failed to augment, and actually decreased granule cell sIPSC frequency in the presence of the glutamate receptor antagonists, kynurenic acid (3 mM) and APV (20 µM) (Fig 9E, sIPSC frequency in Hz, KyA+APV: 0.18±0.02; +500 nM THIP 0.08±0.02, n = 6 cells from 3 mice, p<0.05 by paired *t*-test). The interaction between the effect of THIP and glutamate receptor antagonists on granule cell sIPSC frequency in WT mice was statistically significant (Fig 9E, F(1,23) = 24.89, p<0.05 by two-way repeated measures ANOVA; post hoc pairwise comparison using Holm-Sidak method revealed p<0.05 for effect of THIP on sIPSC frequency both in control ACSF and in KyA+APV, p<0.05 for sIPSC frequency in ACSF vs. KyA+APV in THIP and p>0.05 for sIPSC frequency in control ACSF vs. KyA+APV in the absence of THIP). In contrast to the effect on sIPSC frequency, THIP (500 nM) enhanced the amplitude of tonic GABA currents both in ACSF (Fig. 9F, tonic GABA current amplitude in pA, ACSF: 17.2±3.2; 500 nM THIP: 40.8±6.7, n = 8 cells from 3 mice, p<0.05 by paired *t*-test) and in glutamate receptor antagonists (Fig. 9F, tonic GABA current amplitude in pA, KyA+APV: 15.4±2.2; 500 nM THIP: 37.2±4.3, n = 8 cells from 3 mice, p<0.05 by paired *t*-test). Glutamate receptor antagonists did not abolish the THIP-induced increase in granule cell tonic GABA currents (Fig 9F, effect of glutamate receptor antagonists on tonic GABA current amplitude was not significant, F(1,27) = 0.06, p>0.05 by two-way repeated measures ANOVA). Although THIP increased granule cell sIPSC frequency in both rats and WT mice (Fig. 5, 9F), THIP (1 µM) failed to increase sIPSC frequency in *Gabrd*–*/*– mice (Fig. 9G, sIPSC frequency in Hz, ACSF: 0.64±0.32; THIP: 0.39±0.18, n = 5 cells from 2 mice, p>0.05 by paired t-test). Granule cell sIPSC frequency was not different between WT and *Gabrd*–*/*– mice (sIPSC frequency in Hz, WT: 0.19±0.04, median = 0.33, IQR = 0.26–0.38 in n = 6 cells from 3 mice; *Gabrd*–*/*–: 0.64±0.32, median = 0.47, IQR = 0.13–1.17 in n = 5 cells from 2 mice, p>0.05 by Mann-Whitney Rank Sum Test). Moreover, THIP failed to increase sIPSC frequency in all granule cells from *Gabrd*–*/*– mice, regardless of the pre-THIP sIPSC frequency in that cell, indicating that ceiling effects do not underlie the absence of THIP modulation of sIPSC frequency in *Gabrd*–*/*– mice [4]. Thus, our data reveal a causal role for both GABAR δ subunits and intact glutamatergic transmission in THIP modulation of presynaptic GABA release to granule cells.

Taken together, our data are consistent with ethanol-modulation of granule cell extrasynaptic GABARs contributing to enhancement of synaptic GABA release from Golgi cells. Since pharmacologically distinct compounds which enhance extrasynaptic GABAR currents, mimic effects of ethanol on synaptic GABA current frequency, we suggest that a circuit-based enhancement of synaptic GABA release reinforces the direct postsynaptic effect of these drugs on tonic GABA currents thereby augmenting their depressant actions.

## Discussion

Since the demonstration that extrasynaptic GABARs mediate a tonic form of inhibition that regulates neuronal excitability [3], [49], there has been a growing appreciation of the potential role for extrasynaptic GABARs in regulating higher order network function such as learning and consciousness [1]. Extrasynaptic GABARs are modulated by a diverse group of drugs including anesthetics, neurosteroids, sedatives and alcohol. Here we show that, in addition to their direct effects, modulators of tonic inhibition can increase GABA release from presynaptic cells in the cerebellum. Our study reveals that drugs that augment extrasynaptic GABARs in granule cells contribute to a novel, circuit-based enhancement of synaptic inhibition which likely reinforces the inhibitory efficacy of these agents.

### Circuit mechanism of granule cell inhibitory reinforcement

How can enhancing tonic inhibition in postsynaptic granule cells increase presynaptic Golgi cell firing and synaptic release? Among the six major cell types in the cerebellar cortex, including the GABAergic Purkinje neuron, granule cells are the sole glutamatergic cell-type and the only neuron to express the GABAR α6 and δ subunits, which underlie cerebellar tonic inhibition [23], [50]. The ascending and parallel fiber axons of granule cells provide the only excitatory projection within the cerebellar circuit and make functionally equivalent synapses on Purkinje neurons [51]. Remarkably, several recent studies have demonstrated that GABA increases parallel fibers excitability and enhances glutamate release [52]–[55], indicting that granule cell axons express GABARs and that a depolarizing chloride gradient is present within the axon (Fig. 1A). The depolarizing chloride gradient in granule cells may be unique to the axon since blocking GABA receptors has been shown to enhance granule cell firing [3]. Golgi cells are the primary source of synaptic inhibition to granule cells [47], [48]. Although Golgi cells are traditionally believed to receive robust spontaneous synaptic inhibition from molecular layer interneurons (MLI), recent evidence challenges this view and demonstrates that lateral connections from neighboring Golgi cells are the predominant source of their synaptic inhibition [33]. It is likely that Golgi cells receive direct excitatory inputs from both ascending and parallel fiber axons of granule cells. Since there is ample evidence for GABA-mediated increases in glutamate release from parallel fibers [53]–[55], and we find that GABAR agonists increase sEPSC frequency in Golgi cells (Fig. 8), the simplest interpretation of our results is that GABAR agonists increase Golgi cell firing as a consequence of their direct depolarizing action on parallel fibers (Fig. 1A).

We have tested key predictions deriving from the circuit hypothesis. First, since granule cells are the only class of cerebellar neuron with extrasynaptic GABARs and glutamatergic output, if modulation of granule cell GABA currents underlies the changes in cerebellar circuit activity we predicted that GluR antagonists would eliminate ethanol and THIP-induced enhancement of sIPSC frequency (Fig. 1B). Our data confirm that ethanol and THIP fail to increase granule cell sIPSC frequency in GluR antagonists (Fig. 2, 4 and 9), demonstrating that granule cell output contributes to increased Golgi cell synaptic release. Crucially, GluR antagonists did not eliminate ethanol-potentiation of granule cell tonic GABA currents (Fig. 4 and [9], [14], [15], [18]), demonstrating that enhancement of granule cell extrasynaptic GABA currents lies upstream of changes in synaptic GABA release. Recent studies suggest that synaptic spillover does not augment cerebellar tonic inhibition [56]. However, we observed a small, albeit not statistically significant, decrease in ethanol-potentiation of tonic GABA currents in GluR antagonists (Fig. 4C) suggesting a possible reinforcement of tonic inhibition by increases in ambient GABA following enhanced synaptic release.

Second, if changes in GABAR activity on granule cell parallel fibers contribute to enhancement of Golgi cell activity, their effects will be reduced or abolished in GABAR antagonists (Fig. 1C). Notably, ethanol-potentiation of Golgi cell firing was significantly reduced in GABAR antagonists (Fig. 7) confirming that changes in GABAergic inhibition contribute to ethanol-enhancement of Golgi cell firing. However, ethanol also caused a smaller increase in Golgi cell firing in GABAR antagonists (Fig. 7C), consistent with direct ethanol-modulation of Golgi cell excitability [20]. It is intriguing that there is direct ethanol-modulation of Golgi cell excitability in GABAR antagonists (Fig. 7C), yet there is no ethanol-enhancement of sIPSC frequency in GluR antagonists (Fig. 2 & 4) and in *Gabrd*–*/*– mice (Fig. 9). One possibility is that blocking GABARs in Golgi cells, which have robust synaptic inhibition [31], alters their rheobase and renders Golgi cells more responsive to modulation of membrane currents than under conditions of normal synaptic inhibitory tone. While ethanol has been shown to decrease glutamateric synaptic currents [57]–[59] at certain central and peripheral synapses, we find that THIP, a selective modulator of extrasynaptic GABA receptors, enhances the frequency of Golgi cell sEPSCs (Fig. 8) consistent with the circuit hypothesis. Finally, if extrasynaptic GABARs in granule cells underlie all downstream circuit effects, selective elimination of extrasynaptic GABARs should prevent increases in presynaptic GABA release by extrasynaptic GABAR agonists (Fig. 1C- Orange X). As predicted, ethanol and THIP fail to increase presynaptic GABA release to granule cells in *Gabrd*–*/*– mice (Fig. 9). If depolarizing extrasynaptic GABARs in parallel fibers contribute to ethanol-enhancement of Golgi cell excitability, lack of ethanol effects on synaptic inhibition in *Gabrd*–*/*– mice together with the differential effects in rats with α6 subunit polymorphism suggests that GABARs with δ and α6 subunits are expressed in parallel fibers. Although GABA mediated enhancement of parallel fiber excitability is preserved in *Gabrd*–*/*– mice [55], it is possible that deletion of GABAR δ subunits leads to compensation by GABAR subunits which maintain the GABAergic depolarization of parallel fibers but are not modulated by ethanol or THIP. While parallel fibers appear to express GABAR α1 subunits [53], whether they express GABAR α6 and δ subunits is yet to be determined. Additionally, it is possible that parallel fibers express GABARs distinct form the granule cell soma and dendrites [60]. Regardless of the subcellular location of the extrasynaptic GABARs, the lack of ethanol and THIP effects on granule cell synaptic inhibition in *Gabrd*–*/*– mice confirm that GABAR δ subunits contribute to enhanced Golgi cell GABA release and validate the circuit hypothesis.

### Mechanisms underlying ethanol-inhibition of cerebellar granule cells

Although it is widely accepted that ethanol augments granule cell synaptic inhibition, there are differing opinions on two key elements of the underlying mechanisms [15], [18], [44], [61]. The first concerns the ability of millimolar concentrations of ethanol to directly enhance extrasynaptic GABARs. Studies in recombinant systems [18], [40]–[43] and diverse brain regions have identified that ethanol augments tonic GABA currents mediated by δ subunit-containing GABARs [16], [17], [62]–[64]. However, Borghese et al. [65] did not detect ethanol-enhancement of GABA currents in extrasynaptic receptor isoforms in expression systems. Similarly, Botta et al., [45] were unable to replicate earlier studies demonstrating that a single subunit polymorphism in GABAR α6 subunits leads to genotype specific increases in ethanol-effects on granule cell tonic inhibition [18]. Based on the above results, some studies have called into question the direct ethanol-effects on granule cell tonic inhibition [61]. Our study demonstrates that ethanol and THIP increase tonic GABA currents even in the absence of increases in synaptic GABA release (Fig. 4, 9E&F) confirming direct ethanol-modulation of tonic GABA currents. Moreover, ethanol failed to enhance synaptic GABA release in *Gabrd*–*/*– mice (Fig. 9), validating our assertion that modulation of extrasynaptic GABARs is central to ethanol-effects on granule cell synaptic inhibition.

A related debate concerns the causal mechanism of ethanol-enhancement of GABA release from Golgi cells. In addition to differences in tonic inhibition, there appear to be genotype-dependent differences ethanol-enhancement of synaptic GABA currents in rats with the GABAR α6 subunit polymorphism [18]. Since GABAR α6 subunits are present only in granule cells [23], these findings indicate that modulation of α6 containing extrasynaptic GABARs within granule cells contributes to changes in presynaptic GABA release. Alternatively, recent studies have shown direct ethanol-enhancement of Golgi cell firing [15], [20] and attributed ethanol actions on granule cell tonic and synaptic inhibition to direct ethanol-modulation of Golgi cell activity. In spite of the potential decrease in Na/K pump activity in our recordings at room temperature which could have reduced the direct ethanol effects on Golgi cell firing observed at 32–33°C (Botta et al., 2010), our data confirm the direct effect of ethanol on Golgi cells in GABAR antagonists (Fig. 7A–C). However, unlike earlier studies in which both glutamate and GABA receptors were blocked preempting examination of circuit effects (Botta et al., 2010), our data demonstrate a significant reduction in ethanol-induced potentiation of Golgi cell firing in the presence of GABAR antagonists, underscoring the importance of circuit based effects. Additionally, in spite of direct effects of ethanol on Golgi cell firing in GABAR antagonists (Fig. 7 and [20], [21]), neither ethanol nor THIP increased granule cell sIPSC frequency in GluR antagonists (Fig. 2, 4, 9E) and in *Gabrd*–*/*– subunits mice (Fig. 9C, G). Moreover, recent demonstration that extrasynaptic GABARs in cerebellar granule cells undergo desensitization and show limited potentiation during synaptic GABA spillover [56] suggest that increases in presynaptic GABA release alone may not account for the robust ethanol-enhancement tonic of GABA currents. Thus, our data indicate that, in the presence of intact synaptic transmission, the primary mechanism underlying ethanol-effects on granule cell inhibition are mediated through modulation of extrasynaptic GABARs in granule cells and the subsequent circuit-based excitation of Golgi cells.

### Neurodepressive agents share a circuit mechanism for reinforcing granule cell inhibition

A diverse class of neuropharmacological agents that alter consciousness and impair motor function, including ethanol, THIP, THDOC and propofol have been shown to enhance tonic GABA currents [8], [66]–[68]. Since the acute behavioral effects of ethanol are characterized by impaired motor coordination, balance and eye movements which involve the cerebellum, its physiological effects have been extensively studied [15], [18], [20], [25], [45], [69], [70]. Our study demonstrates that like ethanol, selective modulators of extrasynaptic GABARs, such as low concentrations of THIP and neurosteroids increase synaptic GABAergic inputs to granule cells. Additionally, we find that THIP augments excitatory synaptic inputs to Golgi cells and enhances Golgi cell firing frequency only in the presence of intact glutamatergic transmission. Moreover, we demonstrate that THIP increases synaptic GABAergic inputs to granule cells in WT mice which do not appear to exhibit the GABAR α6 subunit polymorphism and that this effect requires the presence of intact glutamatergic transmission indicating that the observed circuit mechanism is seen in two species and is not specific for rats harboring α6^100Q^ subunits. Thus, we demonstrate a shared mechanism by which enhancing extrasynaptic GABARs augments presynaptic GABA release under conditions of normal synaptic transmission. Such parallel increases in synaptic and extrasynaptic inhibition would be expected to compound depression of granule cell activity and compromise cerebellar function. While tonic GABA currents in cerebellar granule cells are not potentiated during brief increases in synaptic GABA spillover [56], sustained increases in presynaptic GABA release by neurodepressive agents could increase ambient GABA levels and may have contributed to higher ethanol-enhancement of tonic GABA currents in the absence of GluR antagonists (Fig. 4C). Like ethanol, other neurodepressive agonists of extrasynaptic GABARs may compromise cerebellar function by simultaneously increasing synaptic inhibition. Whether such circuit based reinforcing interactions between tonic and synaptic GABA currents is unique to the cerebellum or may be generalized to circuits with extrasynaptic GABARs remains to be determined. In conclusion, ethanol-modulation of tonic GABA currents increases presynaptic GABA release by mechanisms involving the glutamatergic circuit and extrasynaptic GABA receptors. The circuit effects appear to contribute to ethanol-modulation of granule cell inhibition under physiological conditions. The self-reinforcing increase in granule cell inhibition may constitute a common mechanism by which ethanol and neurodepressive drugs that act on extrasynaptic GABARs alter cerebellar processing and contribute to the deficits in motor coordination.
